# Analyzing Peak-to-Average Power Ratio Characteristics in Multi-Channel Intensity Modulation and Direct Detection Flexible Transceivers Deploying Inverse Fast Fourier Transform/Fast Fourier Transform-Based Processing

**DOI:** 10.3390/s23249804

**Published:** 2023-12-13

**Authors:** Lin Chen, Xiaoyu Huang, Wei Jin, Xinyu Wang, Gang Yang, Mingyang Jiang, Yi Huang, Jianming Tang

**Affiliations:** 1 College of Electronics and Information Engineering, Shanghai University of Electric Power, Shanghai 200090, China; hxy000322@mail.shiep.edu.cn (X.H.); wxy0506@mail.shiep.edu.cn (X.W.); y21205032@mail.shiep.edu.cn (G.Y.); 18963739837@mail.shiep.edu.cn (M.J.); 2 School of Computer Science and Electronic Engineering, Bangor University, Bangor LL57 1UT, UK; w.jin@bangor.ac.uk (W.J.); j.tang@bangor.ac.uk (J.T.); 3 Key Laboratory of Specialty Fiber Optics and Optical Access Networks, Shanghai University, Shanghai 200444, China; huangyi1008@shu.edu.cn

**Keywords:** flexible transceivers, multi-channel aggregation, peak-to-average power ratio (PAPR), intensity modulation and direct detection (IMDD), clipping ratio

## Abstract

Cascaded inverse fast Fourier transform/fast Fourier transform (IFFT/FFT)-based multi-channel aggregation/de-aggregation offers a promising solution in constructing highly desirable flexible optical transceivers for considerably improving optical networks’ elasticity, flexibility, and adaptability. However, the multi-channel aggregation operation unavoidably results in generated signals having high peak-to-average power ratios (PAPRs). To solve this technical challenge, this paper first explores the PAPR characteristics of the corresponding flexible transceivers in optical back-to-back (B2B) and 20 km intensity modulation and direct detection (IMDD) transmission systems, and then numerically investigates the feasibility and effectiveness of utilizing the conventional clipping techniques in reducing their PAPR reductions. The results show that the last IFFT operation size is the primary factor determining the PAPRs rather than the channel count and modulation format. For a given last IFFT operation size, the optimal clipping ratio can be identified, which is independent of channel count. With the identified optimal clipping ratio, when the channel count is >4, every two-channel increase in the channel count can only lead to <1.2 Gb/s decreases in the maximum aggregated signal transmission capacity.

## 1. Introduction

Digital signal processing (DSP) plays a vital role in constructing flexible optical transceivers to considerably improve optical networks’ flexibility, elasticity, and adaptability [1]. In the practical implementations of the highly desirable flexible transceivers, cost-effective DSP-based multi-channel aggregation and de-aggregation techniques are employed to flexibly and dynamically aggregate and de-aggregate multiple independent channels of various line rates according to actual end-user requirements.

For existing DSP-based multi-channel aggregation and de-aggregation techniques, multi-channel aggregation/de-aggregation can be realized with a single inverse fast Fourier transform/fast Fourier transform (IFFT/FFT) operation [2,3,4], a digital filtering operation [5,6,7,8], and code-division multiplexing [9,10]. In comparison with these techniques, the cascaded IFFT/FFT-based multi-channel aggregation/de-aggregation technique [11] can not only potentially operate at an ‘add-as-you-grow’ mode to support adaptive and flexible variations in both channel count and channel line rate but also offer additional physical layer network security and guard band-free and highly spectrally efficient transmissions.

However, for the cascaded IFFT/FFT-based multi-channel aggregation/de-aggregation technique, aggregating multiple independent channels with each channel containing multiple subcarriers unavoidably leads to high peak-to-average power ratios (PAPRs). High PAPRs not only require large dynamic ranges of electrical/optical devices but also impose enhanced sensitivity to optical fiber nonlinearity [12,13,14]. Therefore, it is essential to explore the PAPR characteristics of flexible transceivers incorporating the cascaded IFFT/FFT-based multi-channel aggregation/de-aggregation techniques, in order to further explore simple and effective solutions to considerably reduce PAPRs.

To effectively address the high PAPR issues associated with flexible transceivers incorporating the cascaded IFFT/FFT-based multi-channel aggregation/de-aggregation techniques, in this paper, we identify, for the first time, the key parameters determining the PAPRs of the flexible transceivers and, in a 20 km point-to-point (P2P) intensity modulation and direct detection (IMDD) transmission system, numerically explore the feasibility and effectiveness of conventional clipping technique-induced PAPR reductions as well as their resultant impacts on both transmission performance and maximum achievable aggregated transmission capacity.

The results indicate that for the considered P2P flexible transceivers, the PAPRs are primarily determined by the last IFFT size of the cascaded IFFT operations, which is independent of channel count and modulation format. In comparison with the conventional orthogonal frequency division multiplexing (OFDM) signals, the P2P flexible transceiver-produced signals have ~0.3 dB PAPR reductions due to the frequency–time domain-mixed channel aggregations. For the P2P flexible transceivers, the clipping operation can effectively reduce the PAPRs, and more importantly, the optimal clipping ratio is independent of channel count when the last IFFT operation size is fixed. When using the identified optimal clipping ratio, every two-channel increase in the channel count only gives rise to <1.2 Gb/s reductions in the maximum aggregated transmission capacity when the channel count exceeds 4.

This paper is constructed as follows: The operating principle of the flexible transceivers in P2P transmission systems (P2P flexible transceivers) is presented in Section 2. The PAPR characteristics of the P2P flexible transceivers are explored in Section 3. The flexible transceivers’ optimal clipping ratio and their resulting transmission performances are investigated in Section 4 and Section 5, respectively.

## 2. Principle of the P2P Flexible Transceiver

**Figure 1:**
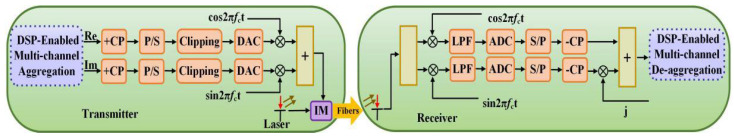
Schematic diagram of P2P flexible transceivers incorporating cascaded IFFT/FFT-based multi-channel aggregation/de-aggregation technique. CP: cyclic prefix. P/S: serial-to-parallel conversion. DAC/ADC: digital-to-analogue/analogue-to-digital conversion. IM: intensity modulator. LPF: low-pass filter. S/P: parallel-to-serial conversion.

**Figure 2:**
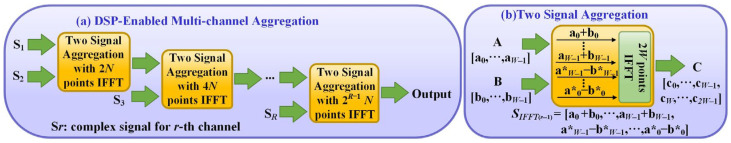
Operating principle of the IFFT-based multi-channel aggregation technique embedded in the P2P flexible transmitter. (**a**) DSP-enabled multi-channel aggregation; (**b**) two signal aggregation.

**Figure 3:**
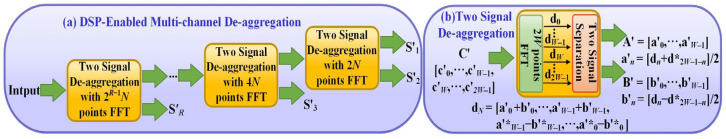
Operating principle of the FFT-based multi-channel de-aggregation technique embedded in the P2P flexible receiver. (**a**) DSP-enabled multi-channel de-aggregation; (**b**) two signal de-aggregation.

The P2P flexible transceivers incorporating a cascaded IFFT/FFT-based multi-channel aggregation/de-aggregation technique are illustrated in Figure 1, and Figure 2 and Figure 3 show the operating principle of the transceiver DSP-embedded IFFT/FFT-based multi-channel aggregation/de-aggregation techniques, respectively. To effectively mitigate the high PAPR issues, DSP-based clipping techniques are implemented after the multi-channel aggregations in the P2P flexible transmitters.

### 2.1. Multi-Channel Aggregation and Clipping Operations in Transmitter DSP

In the transmitter, a complex-valued baseband signal containing *R* independent channels is generated using the cascaded IFFT-enabled multi-channel aggregation technique, as illustrated in Figure 2a. Implementing (*R* − 1) cascaded IFFT operations can aggregate *R* independent channels. To aggregate the *r*-th channel, the required IFFT size for the (*r* − 1)-th IFFT operation is given by

(1) $${L_{IFFT}}_{\left(r-1\right)}=2W=2^{r-1}N,\;\;\;r=(2,\ldots ,R),\;$$

where *N* is the number of data-bearing subcarriers for the first and/or second channels. *W* is the aggregated signal size for the (*r* − 1)-th IFFT operation. Equation (1) indicates that the *r*-th IFFT operation size doubles the size of the (*r* − 1)-th IFFT operation.

As seen in Figure 2b, for the (*r* − 1)-th IFFT operation, the two input signals to be aggregated are *A* = [*a*_0_, *a*_1_, …, *a_W_*_−1_] and *B* = [*b*_0_, *b*_1_, …, *b_W_*_−1_], where signal *A* presents the output of the (*r* − 2)-th IFFT operation, and *B* is the input signal aggregated by the (*r* − 1)-th IFFT operation. The input to the (*r* − 1)-th IFFT operation can thus be expressed as $\;{S_{IFFT}}_{\left(r-1\right)}=\left[a_{0}+b_{0},\ldots ,a_{W-1}+b_{W-1},{a_{W-1}}^{*}-{b_{W-1}}^{*},\ldots ,{a_{0}}^{*}-{b_{0}}^{*}\right]$, where * stands for the conjugate operation. *a_n_* is the *n*-th sample of the output of the (*r* − 2)-th IFFT operation. *b_n_* is the *n*-th sample of the *r*-th channel transmitted signal, which is encoded by an arbitrary modulation format.

After multi-channel aggregation, a complex-valued baseband signal *x_i_*(*t*) is produced. A cyclic prefix (CP) insertion operation, a parallel-to-serial (P/S) operation, two parallel clipping operations, and digital-to-analogue conversions (DAC) are performed successively. The CP insertion and P/S operations are similar to the conventional OFDM CP insertion and P/S operations. Two parallel clipping operations are implemented, respectively, for the real and imaginary parts of the produced complex signal. The employed clipping operation can be expressed as

(2) $$x_{clipping}(t)=\left\{\begin{array}{c} x_{i}(t),\;\;\;\;\;\;\;\;\;\;\;\;\;\;\;\;\;\;\;\;\left|x_{i}(t)\right|\leq A_{max}\\ {\;A_{max},\;\;\;\;\;\;\;\;\;\;\;\;\;\;\;\;\;\;\left|x_{i}(t)\right|>A}_{max}\; \end{array}\right.,$$

where $A_{max}$ is the clipping boundary and $x_{\mathrm{clipping}}\left(t\right)$ is the real or imaginary parts of the clipped complex signal. The resulting clipping ratio is defined as

(3) $$CR=20{log}_{10}(\frac{A_{max}}{E\left[{{|x}_{i}(t)|}^{2}\right]}),$$

where $E\left[\cdot \right]$ denotes the expectation operation.

It should be mentioned that as seen in Figure 1, the analog in-phase and quadrature (IQ) mixers are used to locate the produced baseband complex signal to a desirable radio frequency (RF) spectral region, which can be described as

(4) yit=Re⁡[sit]⊗cos⁡2πfct+Im⁡[si(t)]⊗sin⁡2πfct

where Re[*s_i_*(*t*)] and Im[*s_i_*(*t*)] denote the real and imaginary parts of the produced complex-valued signal *s_i_*(*t*), respectively. *f_c_* represents the central frequency of the sinusoidal signals. Finally, after intensity modulation, an optical signal is produced and then fed into an optical fiber.

For the flexible transceivers, each channel contains multiple independent subcarriers and the number of subcarriers is determined by its corresponding IFFT operation size as presented in Equation (1). The subcarrier modulation format can use M-ary quadrature amplitude modulation (QAM) and/or phase shift keying (PSK), which is similar to conventional OFDM techniques. As such, in generating the subcarriers of each channel, conventional OFDM modulation techniques can be utilized, including the bit-loading techniques for adaptively adjusting each subcarrier’s modulation format and bitrate according to actual use requirements and channel transmission characteristics.

### 2.2. Multi-Channel De-Aggregation Operations in Receiver DSP

As seen in Figure 1, after direct detections, I/Q down-conversion is performed in the receiver. After low-pass filters (LPFs), analogue-to-digital conversion (ADC), serial-to-parallel operations (S/P), and CP removals, the cascaded FFT-based multi-channel de-aggregation operation is then performed in the receiver DSP.

As illustrated in Figure 3a, separating *R* channels requires implementing (*R* − 1) two-signal de-aggregation operations. Each two-signal de-aggregation requires a single FFT operation. To separate the r-th channel, as depicted in Figure 3b, the input of the corresponding two-signal de-aggregation operation is *C*′ = [*c*′_0_, …, *c*′*_W_*_−1_, *c*′*_W_*, …, *c*′_2*W*−1_], and after the FFT operation, the signal *D* = [*d*_0_, …, *d_W_*_−1_, *d_W_*, …, *d*_2*W*−1_] can be obtained. At its output, the two signals of *A*′ = [*a*′_0_, …, *a*′*_W_*_−1_] and *B*′ = [*b*′_0_, …, *b*′*_W_*_−1_] are achieved. Signal *B*′ is the received signal transmitted over the *r*-th channel, and signal *A*ʹ is the input to the (*r* − 1)-th two-signal de-aggregation operation. Detailed principles of the cascaded IFFT/FFT-based multi-channel aggregation/de-aggregation technique can be found in [11].

In the cascaded IFFT/FFT-based multi-channel aggregation/de-aggregation process, each IFFT (FFT) operation will aggregate (separate) a new channel into (from) the transmitted (received) signal. Such multi-channel aggregation and de-aggregation process is independent of signal modulation formats. Due to IFFT/FFT operation features, the aggregated multiple independent channels are multiplexed in both the time domain and the frequency domain, thus offering additional physical layer network security [11]. By activating/de-activating the corresponding IFFT/FFT operations, an arbitrary number of independent channels, each tailored to a specific application/service, can be aggregated and de-aggregated. Detailed explanations of the flexible transceiver-adopted IFFT/FFT-based multi-channel aggregation/de-aggregation process can be found in [11].

## 3. PAPR Characteristics of P2P Flexible Transceivers

For the P2P flexible transceivers, multiple channels are aggregated, and each channel contains multiple subcarriers. This leads to relatively high PAPRs of the produced signals.

**Figure 4:**
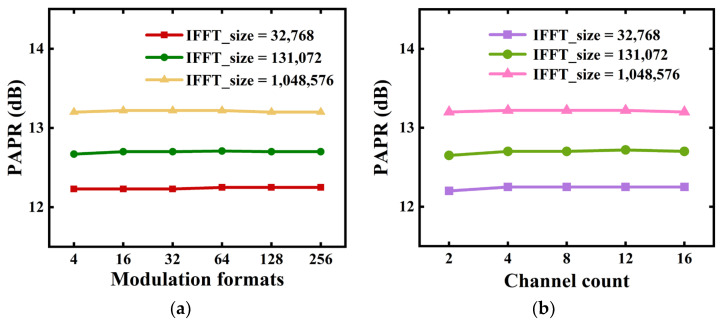
PAPR characteristics of the P2P flexible transceivers for (**a**) various signal modulation formats (4 independent channels) and (**b**) different channel counts (adopting 16 QAM).

To extensively explore PAPR performances associated with the P2P flexible transceivers, PAPRs at a complementary cumulative distribution function (CCDF) of 10^−3^ are plotted in Figure 4.

To simplify the PAPR calculations, we only consider three different sizes of the last IFFT operations, which are fixed at 32,768, 131,072 and 1,048,576. In calculating Figure 4a, the aggregated channel count is fixed at 4. It shows that when the last IFFT size is fixed, the resulting PAPRs are independent of the subcarrier modulation format. In addition, the last IFFT operation with a large IFFT size leads to a relatively high PAPR.

In obtaining Figure 4b, 16 QAM was adopted, while the aggregated channel counts varied from 2 to 16. The results show that when the last IFFT operation size is fixed, the signal PAPRs are independent of the aggregated channel counts.

The results in Figure 4 indicate that for the P2P flexible transceivers, the PAPRs are determined only by the size of the last IFFT operations, which are independent of the subcarrier modulation format and channel count.

## 4. Optimal Parameter Identification

The digital domain clipping operations offer a simple solution to effectively mitigate the high PAPRs of the P2P flexible transceivers. A relatively low (high) clipping ratio leads to relatively large signal distortions (small PAPR reductions). As such, optimal parameter identifications are necessary. In this section, special attention is thus given to the explorations of optimal clipping ratios and DAC/ADC quantization bits.

### 4.1. Clipping Operation-Induced PAPR Reductions

**Figure 5:**
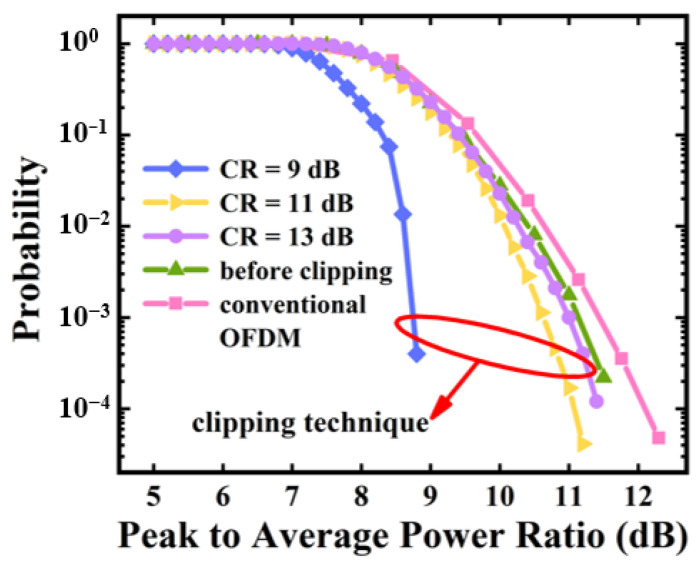
CCDF curves of flexible transceiver-produced baseband complex signals before and after clipping.

The CCDF curves of the P2P flexible transceiver-produced baseband complex signals before/after the clipping operations are plotted in Figure 5, respectively. The last IFFT size is 1024, and the produced complex signals contain four independent channels. For comparisons, the CCDFs of the conventional complex OFDM signals with IFFT sizes of 1024 are also presented in the same figure. The modulation formats are fixed at 16 QAM for all the considered cases.

**Figure 6:**
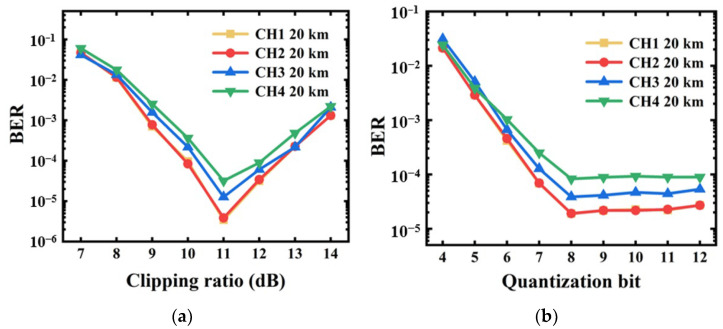
The 20 km transmission performances for (**a**) different clipping ratios and (**b**) quantization bits.

As shown in Figure 5, it can be found that compared with the conventional OFDM signals, the P2P flexible transceiver-produced signals have ~0.3 dB reductions in PAPRs, which mainly arise from the multi-channel aggregations in both the frequency domain and the time domain. For the P2P flexible transceivers, the clipping operations can effectively reduce PAPRs and a small clipping ratio leads to low PAPRs. For the optimal clipping ratios of 11 dB (identified in Figure 6a), the clipping-operation-induced PAPR reductions are >0.5 dB.

### 4.2. Optimal Clipping Ratio and Quantization Bits

To maximize the achievable transmission performance of the P2P flexible transceiver, the optimal clipping ratio and quantization bit of the DAC/ADCs are identified. The impacts of variations in clipping ratio and quantization bit on the P2P flexible transceivers transmitting four independent channels over 20 km standard single-mode fibers (SSMFs) are, respectively, explored and shown in Figure 6a,b. The key parameters of the 20 km IMDD transmission systems are given in Table 1.

In calculating Figure 6a, a quantization bit of 8 is considered. The received optical power is −2 dBm. As shown in the figure, the bit error ratios (BERs) of all the considered channels reach the lowest value at the optimal clipping ratio of 11 dB. For the clipping ratios of <11 dB, a decreasing clipping ratio leads to considerable signal distortions, thus resulting in nonnegligible BER performance degradations [15]. In contrast. when clipping ratios are >11 dB, as discussed in Section 5.2, insufficient signal clipping results in high PAPRs and large carrier-to-signal power ratios (CSPRs), thus finally leading to effective signal-to-noise ratio (SNR) reductions and BER performance degradations.

The quantization bit-dependent BER performances are shown in Figure 6b. In obtaining the result, the optimal clipping ratio of 11 dB is used and the received optical power is fixed at −2 dBm. It shows that when the quantization bit is <8, a low quantization bit leads to high BERs for all the channels due to the low quantization-bit-induced quantization noise effects. For a quantization bit >8, the BER performance improvements are negligible. This implies that for the P2P flexible transceivers, the optimal quantization bit is 8, which is sufficient for mitigating quantization noise effects.

## 5. Transmission Performances

This section thoroughly explores the performances of the P2P flexible transceivers over 20 km IMDD transmission systems with the key parameters listed in Table 1. The optimal clipping ratio of 11 dB and the optimal DAC/ADC resolution of 8 bits are employed.

### 5.1. 20 km SSMF Transmission Performances

**Figure 7:**
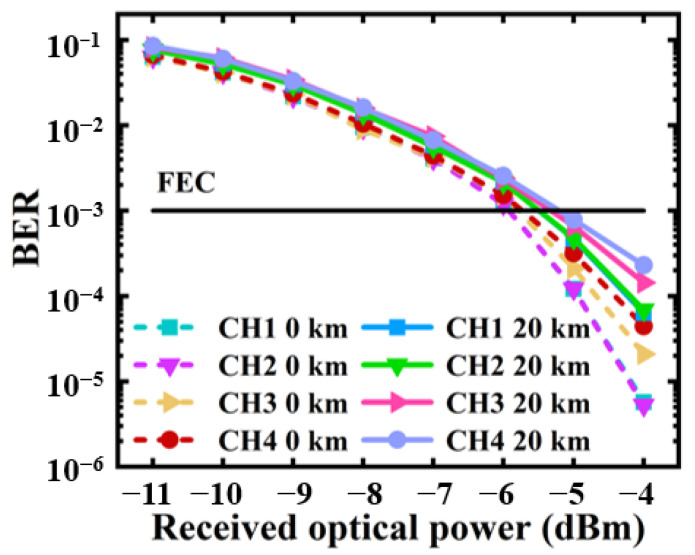
BER performances before and after 20 km fiber transmissions.

The BER performances of all four involved independent channels before and after 20 km SSMF transmissions are investigated and plotted in Figure 7. Here, adaptive bit-loading is used for each channel to achieve the maximum channel transmission performances. Each channel bitrates are listed in Table 2, which give rise to a total bitrate of 68.5 Gb/s over a 12.5 GHz radio frequency spectral region.

As seen in Figure 7, after 20 km SSMF transmissions, due to adaptive bit-loading, all the channels have similar transmission performances. The fiber-transmission-induced power penalties at a BER at the forward error correction (FEC) limit of 1.0 × 10^−3^ are < 0.6 dB for all the considered channels.

### 5.2. Impacts of Clipping on Maximum Aggregated Transmission Capacity

**Figure 8:**
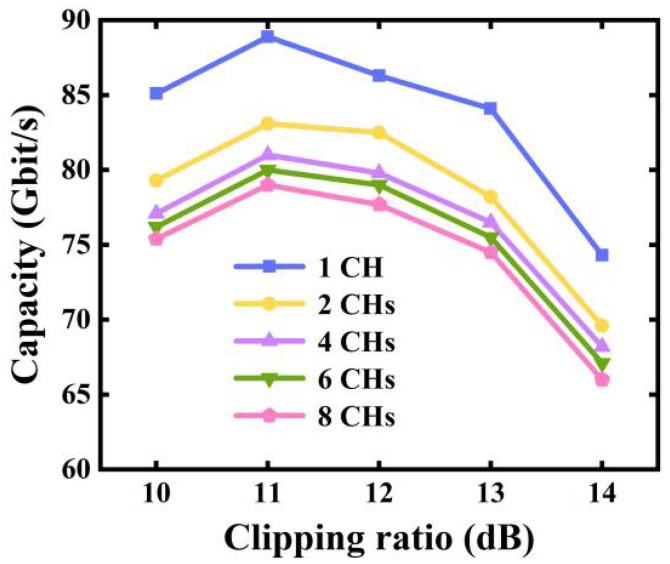
Aggregated multi-channel transmission capacity versus clipping ratio.

Employing the parameters stated in Table 1, for different channel counts, the influences of signal clipping on the maximum achievable aggregated transmission capacity are explored and illustrated in Figure 8.

**Figure 9:**
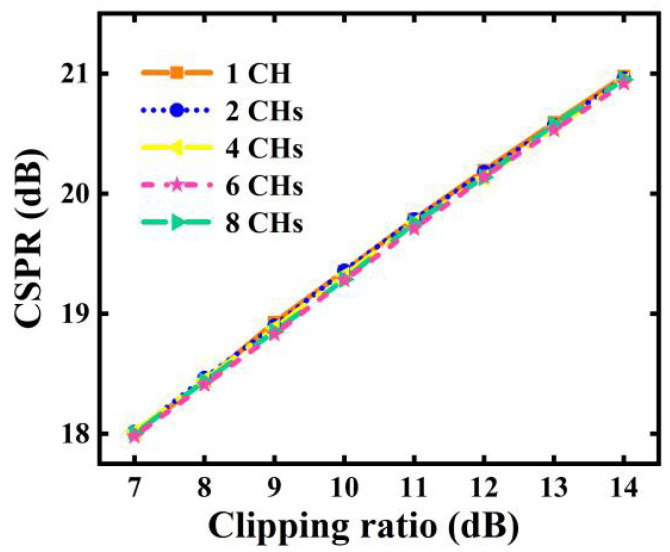
Impacts of clipping ratio on CSPR of produced optical signals containing various channel counts.

As shown in Figure 8, the optimal clipping ratio is 11 dB, which agrees well with the results in Section 4.2. When the clipping ratios are <11 dB, the clipping-induced signal distortions become more pronounced, thus leading to considerable reductions in the aggregated transmission capacity. In contrast, when clipping ratios are >11 dB, insufficient signal clipping can not only lead to high PAPRs but also result in high CSPRs for the produced optical signals, as seen in Figure 9. Such high CSPRs can reduce the SNRs of the received signals [16,17], thus giving rise to considerable BER performance degradations.

**Figure 10:**
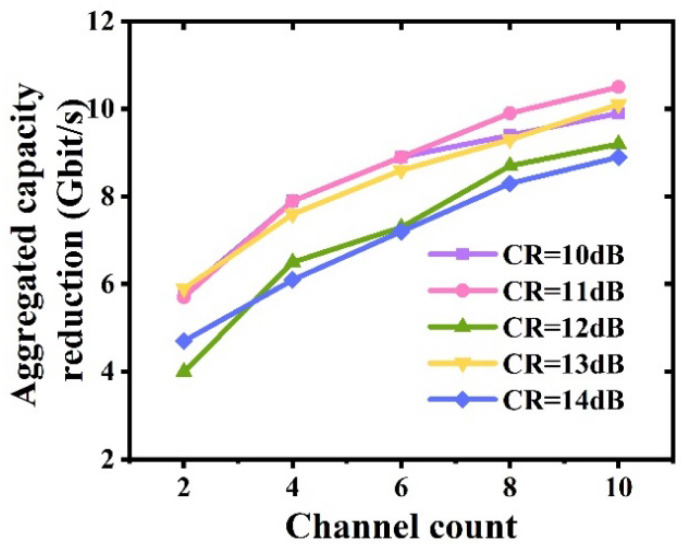
Channel count increase induced reductions in aggregated multi-channel transmission capacity. Here, the transmission capacity in the case of transmitting only one channel is the benchmark.

It is also interesting to point out that when the last IFFT operation size is fixed, the optimal clipping ratio is independent of channel count, as verified in Figure 8. As seen in Figure 10, with the identified optimal clipping ratio, when the channel count increases, the maximum aggregated transmission capacity is slightly reduced because of clipping-induced channel interferences [1], and it can also be found that every additional two-channel increase in the channel count can only lead to <1.2 Gb/s reductions in the aggregated capacity when the channel count is >4. As illustrated in Figure 9, when the size of the last IFFT operations is fixed, clipping ratio has a linear relationship with CSPR, which is independent of channel count.

The impacts of clipping on the BER performances of the P2P flexible transceivers over 20 km SSMFs are illustrated in Figure 6. It can be seen from Figure 5 and Figure 6 that clipping can effectively reduce PAPRs by introducing signal distortions [18]. Such signal distortions can result in unwanted channel interferences [1,11], which can be further mitigated by adopting clipping noise cancellation techniques [19].

## 6. Conclusions

This paper has explored the PAPR characteristics of the flexible optical transceivers incorporating the cascaded IFFT/FFT-based multi-channel aggregation/de-aggregation techniques in optical B2B and 20 km IMDD transmission systems and further numerically investigated the conventional-clipping-technique-induced PAPR reductions and the resultant impacts on transmission performances and maximum aggregated transmission capacities. The results show that the PAPR is mainly determined by the last IFFT size of the cascaded IFFT operations only, and it is independent of channel count and subcarrier modulation format. For a specific last IFFT size, the optimal clipping ratio can be identified, which is independent of channel count. With the identified optimal clipping ratio, if the channel count is >4, increasing the channel count by an additional two channels can only result in <1.2 Gb/s reductions in the maximum aggregated transmission capacity.

## Figures and Tables

**Table 1:** Transceiver and transmission system parameters.

Parameter	Value
Channel count	4
IFFT/FFT points	256/512/1024
Data-carrying subcarriers	128/128/256/512
Modulation formats	16 QAM
The central frequency of RF signals for up-conversion and down-conversion	6.25 GHz
Cyclic prefix	12.5%
DAC and ADC sample rate	12.5 GS/s
Fiber length	20 km
Optical launch power	8 dBm
PIN detector sensitivity	−19 dBm
PIN responsivity	0.8 A/W
Linear fiber attenuation	0.2 dB/km
SSMF dispersion parameter at 1550 nm	16 ps/(nm·km)
SSMF dispersion slope at 1550 nm	0.2 dB/km
Kerr coefficient	2.35 × 10^−20^ m^2^/W

**Table 2:** Channel bitrate (Gb/s).

CH1	CH2	CH3	CH4
8.5	8.5	17.1	34.4

## Data Availability

Data are contained within the article.
